# Use of medicinal plants for human health in Udzungwa Mountains Forests: a case study of New Dabaga Ulongambi Forest Reserve, Tanzania

**DOI:** 10.1186/1746-4269-3-7

**Published:** 2007-01-26

**Authors:** Rukia A Kitula

**Affiliations:** 1 Department of Wood Utilization, Sokoine University of Agriculture, Morogoro – Tanzania; 2 Institute of Marine Sciences, University of Dar es salaam, P.O. Box 668, Zanzibar, Tanzania

## Abstract

The dependence of local people on plant medicine from natural forests has a long tradition in Tanzania and is becoming increasingly popular among rural and urban communities due to among others increase in living costs. The study on utilization of medicinal plants for meeting heath care needs was carried out between March 2001 and March 2002 in New Dabaga Ulongambi Forest Reserve, Tanzania. The study aimed at generating necessary data for the Udzungwa Mountains Forest Management project to draft sound Joint Forest Management plans. Specific objectives of the study among others were to assess knowledge associated with utilization of medicinal plants for health care needs as well as factors associated in using plant medicines in the study area. A questionnaire survey, market survey and literature review were used to collect information. Tools used for data analysis were Statistical Packages for Social Science and content analysis. A total of 45 plant species were documented curing about 22 human diseases. Medicinal plants were readily available throughout the year and plentiful in the forest reserve. Roots and leaves were the plant parts harvested for medicinal purposes. Processing of plant medicines involved boiling, pounding, soaking in water and chewing. Distance to health facility, income level of the household and beliefs contributed to the use of plant medicines. The study concluded that medicinal plants play an important role in providing primary health care to the rural communities. It is recommended that in achieving joint forest management (JFM), villagers adjacent to the forest reserve should be sensitised on the importance of JFM through seminars, workshops, drama, school songs or video show. During the development of a joint draft management plan, villagers as an informal institution must define their priority needs of use of parts of the forest in collaboration with the Udzungwa Mountains Forest Management project.

## Background

The loss of habitat is the major factor contributing to the depletion of natural resources in Tanzania. Among the natural resources are medicinal plant species that are gathered from the wild. It is estimated that over 80% of rural people in Tanzania depend on traditional healers and herbs for their primary health care needs [1] and [2]. Reliance on medicinal plants creates the need to maintain and conserve biodiversity. Thus, management of medicinal plant species can not be seen separately from general forest management. Also managing the forest for medicinal plant species is more sustainable from ecological and social perspectives.

Collection of medicinal plants for use and export is extremely detrimental to certain species. Popular but slow growing and or naturally rare plant species are often under pressure [3]. Medicinal plants are threatened by man's activities, since these plants have several other useful applications, for example, timber, fuel, and construction poles. Sustainable management of medicinal plant species is important, not only because of their value as a potential source of new drugs but also due to reliance on medicinal plants for health care and increase of income to the household.

Conservation of medicinal plants, especially endangered ones depend largely on the conservation of the ecosystem in which they occur. Due to the increased rate of over exploitation of the natural resources in Tanzania, the government realized that in order to have sustainable utilization of natural resources local people must be involved in the management of their natural resources. The major question is how to organize people to sustainably manage the forest in a manner that can contain available resources for future use. This issue faced the Udzungwa Mountains Forest Management (UMFM) project. The project faced a problem of how to sustain the available resource in the forest reserves. Hence, the study on utilization of medicinal plants for heath care needs was carried out in the New Dabaga Ulongambi Forest Reserve (NDUFR), Tanzania. The study aimed to generate necessary data for the UMFM project to draft sound Joint Forest Management (JFM) plans. Specifically, the study assessed the knowledge associated with utilization of medicinal plants for health care needs and factors associated in using medicinal plants in the study area.

## Methods

Data were obtained through interviews using both structured and semi-structured questionnaires, market survey, and reviewing both published and unpublished documents. Purposive sampling was used to interview all herbalists, health workers and traditional midwives in each village located in six villages surrounding the NDUFR namely Kidabaga, Magome Isele, Lulanzi, Lusinga and Ilamba. The sample involved thirty herbalists, six health workers and twenty TMs. Local authorities were used to identify and locate the locally recognized required respondents in each study village. However, random sampling design was used to select households for non medicinal plants specialists to be interviewed in each village visited. A total of thirty-six non medicinal plants specialists were interviewed. Content analysis was used for analysing qualitative data. Data collected through structured instruments were analyzed by using the Statistical Package for Social Sciences (SPSS) computer program. Scientific identification of the medicinal plants was done at the Arusha National Herbarium, Tanzania.

## Results and discussion

### Medicinal plant and diseases treated

A total of 45 plant species found in the NDUFR are used as plant medicines (Table 1). The number of medicinal plants recorded in this study is lower than the 295 medicinal plants reported by [4] for the Bwindi Impenetrable Forest, Uganda. The number is also lower than that reported by [5] and [6] for East Usambara and Ruvu Forest Reserve, Tanzania respectively in which each documented 185 species. This suggests that the forest has fewer number of medicinal plants compared to the other forests. Traditional medical practitioners (TMPs) reported that medicinal plants are readily available throughout the year. They often collect plant medicine from the forest reserve illegally because the forest is closed.

**Table 1:** List of complications cured by plant medicine from NDUFR

**Complication cured**	**Species name**	**Local names**	**Part(s) used**	**Process**	**Application**
Abdominal pain	*Rhamnus mucronata*	Kihanga, Kihaga	Root	Boil	Drink
	*Zanha africna*	Kiwangaduma	Root	Boil, pound	Drink, sniff
	*Ocimum suave*	Lwenyi	Root	Boil	Drink
	*Embelia schimperi*	Mnyainyai	Leaves, root	Chew, boil	Swallow, drink
	*Nuxia floribunda*	Mngogo	Root	Pound, boil	Drink
Aphrodisiac	*Faurea saligna*	Lwendi	Root	Boil	Drink
	*Nuxia floribunda*	Mngogo	Root	Boil	Drink
	*Sonchus schweinfurthii*	Sungasunga	Root	Boil	Drink
Back ache after delivery	*Ocimum suave*	Lwenyi	Leaves	Pound mix with sheep tail fat	Insert to the anus
Boil on breast during breastfeeding	*Maesa lanceolota*	Mhenyi	Bark	Pound	Boil, smear on breast
	*Ficus sp*.		Bark	Pound	Boil, smear on breast
Conception	*Rhamnus mucronata*	Kihanga, Kihaga	Root	Boil	Drink
	*Casearia gladiiformis*	Mlelulelu	Root	Boil	Drink
	*Scolopia stolzii*	Mgogola	Root	Boil	Drink
Convulsion	*Ziriphus sp*.	Kitanula	Leaves	Pound	Drink and apply the whole body
	*Clausena anisata*	Mnung'anung'a	Leaves	Pound	Drink and apply the whole body
	*Bersama abyssinica*	Mnyatoma	Leaves	Pond	Drink and apply the whole body
	*Zanha africna*	Kiwangaduma	Leaves	Pound	Drink and apply the whole body
	*Rubus sp*.	Mwifya	Leaves, root	Pound	Drink
Cough	*Rhamnus mucronata*	Kihanga, Kihaga	Root	Boil	Drink
	*Citrus aurantifolia*	Mdimu	Fruit, leaves	Cut, boil	Drink
	*Osyris lanceolata*	Mdunula			Drink
Diarrhoea	*Olea europea*	Mlyandege	Root	Boil	Drink
	*Protea chionantha*	Nwinyigi	Root	Boil	Drink
Epilepsy	*Rubus sp*.	Mwifya	Leaves, root	Pound	Drink
Headache	*Zanha africna*	Kiwangaduma	Root	Boil, pound	Drink, sniff
Heart burn	*Embelia schimperi*	Mnyainyai	Leaves, root	Chew, boil	Swallow, drink
Irregular menstruation period	*Nuxia floribunda*	Mngogo	Root	Pound, boil	Drink
Malnutrition	*Datura culeastrum*	Lidasi	Fruit	Cut, squeeze, boil	Insert to the anus so as to diarrhoea
	*Solanum incanum*	Ndulandula	Fruit	Cut, squeeze, boil	Insert to the anus so as to diarrhoea
Measles	*Sonchus schweinfurthii*	Sungasunga	Leaves	Pound	Apply to the whole body
Pneumonia	*Embelia schimperi*	Mnyainyai	Leaves, root	Chew, boil	Swallow, drink
Skin rashes	*Datura culeastrum*	Lidasi	Leaves	Pound	Apply to the infected areas
	*Ocimum suave*	Lwenyi	Leaves	Pound	Apply to the infected areas, drink
Snake bite	*Bersama abyssinica*	Mbasamono	Leaves	Pond	Drink and apply to the bitten area/whole body
Venereal disease	*Ficus sp*.	Msombi	Bark	Pound	Boil, smear on breast
	*Ozorora insignis*	Mwitapozi	Root	Boil	Drink
Vomiting	*Cyphostema sp*.	Mtogonigo	Leaves	Pound, boil	Drink
Worm infestation	*Cyphostema sp*.	Mtogonigo	Root	Boil	Drink
	*Ocimum suave*	Lwenyi	Leaves	Pound	Apply to the infected areas, drink
	*Ozorora insignis*	Mwitapozi	Root	Boil	Drink

### Gender and medicinal plants collection

All TMs in the surveyed villages were found to be females and acquired skills by inheritance from their elders. Some got training from health centres for the purpose of improving their services. Male herbalists were involved in midwifery issues in case of difficult deliveries or prescribing medicinal plants. These results are similar to those reported by [7]. This suggests that the work is segregated by sex, and is the way the knowledge of plant medicine utilization is passed to young generation contributes to this segregation. However, the male (70%) herbalists were observed to be more frequent collectors than female herbalists (30%). The high frequency of male plant collectors was attributed to the nature of collection sites, as most medicines were not found close to the village.

### Plant part harvested

The survey indicated that the most commonly harvested plant parts were roots (44.3%) followed by leaves (23.6%) and fruits (11.8%). This is because it is believed that roots contain more concentration of the active ingredients. The least harvested plant part was barks (0.9%) and stems (0.5%). These results are in agreements with the findings by [8] and [9] who reported that in Mwingi District, Kenya and Bagamoyo District, Tanzania respectively, roots are the most commonly harvested plant part for medicinal purposes for maternal and child health.

### Processing methods

Boiling (50%) and pounding (40%) were the common methods used for preparing plant medicines before administering to sick people. Boiling was believed to be efficient in extracting active ingredient and for hygienic reasons. These results are similar with those reported by [6] and [9]. Most of the mentioned medicinal plants were used singly. In some cases herbalist prepared a mixture of two or more plant medicines to treat a single disease (Table 1).

### Dosage and side effects

The study revealed that dosages of plant medicine were not specific and their side effects were not known. This is dangerous because it is possible to overdose oneself with the remedy without knowing. However, the quantity of medicinal plants used per patient as reported by various herbalists depends on the concentration (colour change) after processing, type of disease and age of the patient. The most common unit of measure in the liquid form for children under five years old was one teaspoon to one-quarter a cup while adult was one cooking spoon to one cup.

### Marketing

There was no formal market in the study villages for selling plant medicine. Vendors of traditional medicine were surveyed in Iringa town market. It was observed that both women and men who are TMPs sell at the Iringa town market roots, barks and powdered plant materials packed into small bottles at Tsh. 300 to Tsh. 7000. The vendors are capable of realising a monthly income between Tsh. 150,000 to Tsh. 200,000 from the sale of plant medicines. This means that a considerable amount of income is made from selling traditional medicine.

### Factors associated to the use of plant medicine

#### Access to health facilities and medication charges

The results revealed that there was an inadequate number of dispensaries and health workers for maternity care (only three dispensaries were built). The ratio of doctor to patient in the study village is 1: 540. Health centers were built in three villages namely Kidabaga, Magome and Kilolo. All six villagers use these centers and people from Isele, Lulanzi, Lusinga and Ilamba villages have to walk a long distance (up to 7 km) to reach a health center. Discussions with Rural Medical Aids revealed that the dispensaries were not stocked with basic medications and facilities necessary for maternity care. At Kidabaga dispensary there were 14 hospital beds, in which 4 beds were in a male ward while 10 beds in the female ward whereas at Magome dispensary there were only two beds. For many years, people have had free access to medical services and the number of patients attending to health centres was 20 per day. Since the introduction of cost sharing on medication in 1993 the number of patients attending health centres declined from 20 to 5 patients per day. Patients are charged Tsh. 1000 as a registration fee for treating emergency diseases or Tsh. 5000 per year as a registration fee for treating a family (that is, father, mother and children). Furthermore, transport facility was not easily available and the terrain of the area was bad especially during the rainy season. Hence, resorting to the use of medicinal plants, which were easily accessible and cheap is an important component to health care. Likewise, traditional medical practitioners were readily available.

#### Income level of the household

It was observed that about 90% of the respondents had average annual revenue of about Tsh. 250,434.78. Nevertheless, the price charged by the TMPs for treating a single disease ranged from Tsh. 200–3000. The price range depends on the type of disease, season, and availability of medicinal plants. Moreover, it was observed that many diseases of mothers and children were treated free of charge. In addition, some of the herbalists preferred to be paid after disease recovery. This illustrates that the use of traditional medical practitioners is cheaper compared to the costs for conventional treatment.

#### Taboos and beliefs on plant medicines utilization

The use of plant medicine in the study area was associated with a belief in the power of medicinal plants to bring good health during pregnancy and child growth, enhancing conception, preventing damage from evil eyes and witchcraft, as contraceptives, and to induce and provide for easy labour.

## Conclusion

In conclusion, medicinal plants play an important role in providing primary health care to the rural communities of the Iringa Region. The use of medicinal plants from NDUFR requires adequate control measures to safeguard the future use of these resources. As such it becomes crucial that the JFM be introduced to the area. In achieving JFM, villagers adjacent to the forest reserve should be sensitized on the importance of JFM through seminars, workshops, drama, school songs or video shows. However, during the development of a joint draft management plan, villagers must define their priority needs of use of parts of the forest in collaboration with the Udzungwa Mountains Forest Management project.

## List of abbreviations

JFM – Joint Forest Management

Km – kilometers

NDUFR – New Dabaga Ulongambi Forest Reserve

PRA – Participatory rural appraisal

SPSS – Statistical Package for Social Sciences

TMPs – Traditional medical practitioners

Tsh – Tanzania Shillings

UMFM – Udzungwa Mountains Forest Management
